# Seasonal changes in bird communities on poultry farms and house sparrow—wild bird contacts revealed by camera trapping

**DOI:** 10.3389/fvets.2024.1369779

**Published:** 2024-02-20

**Authors:** Alberto Sánchez-Cano, Maria-Cruz Camacho, Yolanda Ramiro, Teresa Cardona-Cabrera, Ursula Höfle

**Affiliations:** SaBio Research Group, Institute for Game and Wildlife Research IREC (CSIC-UCLM-JCCM), Ciudad Real, Spain

**Keywords:** synanthropic birds, indirect contact, shared diseases, poultry farms, bridge species, biosecurity

## Abstract

**Introduction:**

Wild birds are considered reservoirs of poultry pathogens although transmission routes have not been conclusively established. Here we use camera trapping to study wild bird communities on commercial layer and red-legged partridge farms over a one-year timeframe. We also analyze direct and indirect interactions of other bird species with the house sparrow (*Passer domesticus*), a potential bridge host.

**Methods:**

We conducted camera trapping events between January 2018 and October 2019, in two caged layer farms, one free-range layer farm, and two red-legged partridge farms in South-Central Spain.

**Results and Discussion:**

We observed wild bird visits on all types of farms, with the significantly highest occurrence on red-legged partridge farms where food and water are more easily accessible, followed by commercial caged layer farms, and free-range chicken farms. The house sparrow (*Passer domesticus*) followed by spotless starlings (*Sturnus unicolor*) was the most encountered species on all farms, with the highest frequency in caged layer farms. On partridge farms, the house sparrow accounted for 58% of the wild bird detections, while on the free-range chicken farm, it made up 11% of the detections. Notably, the breeding season, when food and water are scarce in Mediterranean climates, saw the highest number of wild bird visits to the farms. Our findings confirm that the house sparrow, is in direct and indirect contact with layers and red-legged partridges and other wild birds independent of the type of farm. Contacts between house sparrows and other bird species were most frequent during the breeding season followed by the spring migration period. The species most frequently involved in interactions with the house sparrow belonged to the order Passeriformes. The study provides a comparative description of the composition and seasonal variations of bird communities in different types of layer/ poultry farms in Southern Spain i.e. a Mediterranean climate. It confirms the effectiveness of biosecurity measures that restrict access to feed and water. Additionally, it underscores the importance of synanthropic species, particularly the house sparrow, as potential bridge vector of avian pathogens.

## 1 Introduction

In recent years, in large parts of the Northern hemisphere there has been an increasing focus on enhancing animal welfare within the agricultural sector, encompassing poultry production (1, 2). To address these concerns, new production systems have been designed and implemented, providing animals with the opportunity to reside in environments that are more like their natural habitats and less restrictive. However, these innovative production systems, especially those employed in the poultry industry, can result in greater interaction between domestic and wild birds, including their feces (3–5).

Various species of wild birds, known as synanthropic birds, belonging to the families Columbidae, Corvidae, and Passeridae, have demonstrated a remarkable adaptation to exploit resources generated by human activities, such as food, water and shelter (6). Examples from these families that include the house sparrow (*Passer domesticus*), the tree sparrow (*Passer montanus*), European starlings (*Sturnus vulgaris*), and feral pigeons (*Columba livia*), can inhabit diverse environments created by humans, from urban areas to isolated farms. Some of these birds, especially the house sparrow easily enter production facilities, through small gaps in exterior walls often even when protected by bird nets (6). Thus, if individuals of such species are in close contact with poultry on one hand and wild bird species such as waterfowl that not usually enter enclosures or barns on the other hand, they could act as so-called bridge hosts in the transmission of pathogens.

In terms of risks in addition to abundance of farm birds, composition of the farm bird community could be important (7). The dilution effect hypothesis postulates that a higher biodiversity is linked to a lower prevalence of pathogens, as species-rich communities harbor individuals in which a specific pathogen cannot multiply to sufficient levels to transmit infection to new susceptible individuals. This reduces the overall success of pathogen transmission and, consequently, the prevalence of pathogens (8).

In the context of pathogens transmitted by wild birds, it is expected that in places where birds congregate in farms, the presence of many different species with diverse susceptibilities would make it more difficult for a pathogen to persist and spread, especially if a single species is the key reservoir for this pathogen. Meanwhile, the presence of species that are migratory on farms could increase the likelihood of the introduction of pathogens that these birds may have encountered on their migratory route. Finally, the risk of pathogen spillback from poultry to wild birds may also vary considerably with the species of wild bird encountering poultry or its feces.

Poultry farms attract wild birds due to water (puddles, canals, ditches) or food resources (spilled feed, drying feed, insects in manure, carcasses). These factors could increase the contacts between wild birds and bridge bird species, as well as increase the abundance of the latter and thus also contact between wild birds and domestic poultry (chickens, turkeys, game birds) (9). This contact can occur directly or indirectly through contamination of resources, thereby increasing the risk of transmission and spillback of avian pathogens, such as avian influenza viruses (AIV), *Salmonella* sp., and avian coronaviruses (10) among others. In this context, European starlings for example are a high-priority species for avian pathogen exposure detection studies as they can form large flocks in livestock feeders during the winter and autumn seasons, representing a potential risk of pathogen incursion into poultry farms, especially during the breeding season (11).

The unforeseen and unprecedented spread and change in the epidemiology of the highly pathogenic avian influenza virus (HPAIV) H5N1 of clade 2.3.4.4b, now fatally affecting new species, new continents, during all seasons, is decimating wild and domestic bird populations in much of the world, especially in the European and American continents (12). In contrast to other HPAIV it shows self-sustained prolonged transmission in wild birds and has already affected many poultry operations globally (12). This increases concerns regarding the potential transmission pathways of AIV by synanthropic bridge species. Migratory waterfowl, considered the main reservoirs for AIV (13, 14), play a key role in the introduction of many AIV subtypes through asymptomatic shedding, exerting a significant factor in the redistribution and transmission of these subtypes to domestic poultry (15). Several studies on the movements of migratory waterfowl have demonstrated their involvement in the large-scale spread of the virus (16). However, it should be noted that due to their ecological needs these wild birds rarely come into direct contact with poultry (17). In this scenario, synanthropic birds such as the house sparrow or the European starling are perceived as potential carriers and transmitters of AIV (18, 19), and could act as bridge both after exposure through direct contact with infected waterfowl, or contaminated environment in shared habitat (20).

Biosecurity protocols on farms rarely comprehensively assess how the virus enters the farm and which farm animals may be carriers of AIV. Despite some experimental evidence of the potential for synanthropic bird species to transmit AIV, there are very few studies dedicated to quantifying wild bird interactions with poultry farms. These studies include research in Australia using camera traps to monitor wild birds on different types of layer and meat chicken farms (21). Another study in the Netherlands quantified wild bird access to a free-range commercial laying hen farm by installing video cameras at a critical point for avian influenza (4). In southwestern France, a study used individual direct observations of wild birds on a free-range duck farm (5). Additionally, a recent study in northwestern Italy employed direct observations and camera traps on turkey and broiler duck farms, as well as laying hen farms (22). A study using satellite transmitter data from radio-marked waterfowl, showing occasional but regular incursions of marked birds onto poultry farm premises (23).

Collectively, these studies evaluate the accessibility of poultry farms for wild birds and identify the house sparrow as one of the most common species on farms due to its resident and sedentary nature. However, knowledge gaps exist regarding the frequency of farm visits by other species, seasonal changes in wild bird communities and characterization of sparrow interactions with other wild birds or even poultry on poultry farms.

The goal of this study was to generate data on seasonal changes of wild bird communities on different types of poultry farms and to investigate contacts of a key bridge species with other bird species that are non-residents on poultry premises and that could potentially lead to the acquisition and transmission of pathogens on to poultry. The latter is based upon the fact that in initial visits we observed a significant presence of house sparrows on poultry farms, commonly sighting them in barns and surrounding crop areas, even entering the barns where layers were housed/flight cages of red-legged partridges. Considering the persistence of AIV in bird feces and the environment (20) it has been confirmed that, under favorable conditions of high humidity and low temperature, AIV can persist in feces for extended periods, even in dry manure (24, 25). We hypothesize that in environments where house sparrows and other birds share resources such as food, water, or resting areas, contact could occur through shared surfaces contaminated by feces. If this occurs the house sparrow could become a potential vector for AIV as well as other pathogens.

For our purpose, we conducted camera trapping on the premises of various commercial layer and red-legged partridge farms in the Castilla-La Mancha region, in south central Spain at different time-points throughout the year corresponding to phenological events in wild bird ecology such as the breeding and wintering season as well as the periods during which migratory species conduct their spring and fall migration. We characterized the wild bird communities observed and used the house sparrow, which is the most abundant resident species, and the species most likely to also enter the layer barns/enclosures/flight cages as a potential bridge species. Hence, we analyzed the direct and indirect contacts of the house sparrow with other wild bird species observed in the camera traps. The collected data were used to quantify wild bird visits and their interactions with the house sparrow.

## 2 Materials and methods

### 2.1 Study area

**Figure 1:**
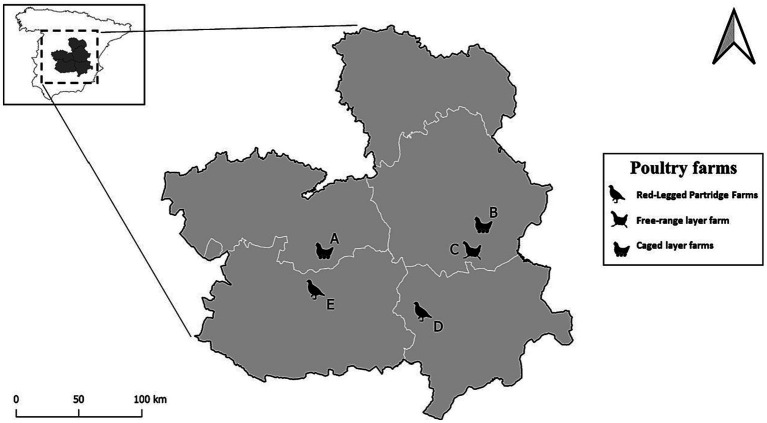
Location of the five farms within the Castilla-La Mancha region in Central Spain, categorized by species and type. The letters (A, B) represent the caged layer farms, the letter C denotes the Free-range layer farm, and the letters (E, D) indicate the red-legged partridge farms.

Our study was conducted in three commercial layer farms and two red-legged partridge farms between January 2018 and October 2019 in south-central continental Spain (Figure 1). The predominant climate in this region is Southern Plateau Continental Mediterranean or according to the Köppen classification Hot summer Mediterranean (26) characterized by mean annual rainfall (mm) from 350 to 550 mm. Mean annual temperature fall between 12 and 15 (°C) and an annual mean temperature range spanning from 18 to 20.5 (°C) (27). The farms under study are not close to large wetlands however the area has a collection of inland temporary wetlands (mostly dry in summer) known as the “Mancha humeda,” which play a crucial role in winter and in the spring and fall migration of wild birds from northern and central Europe to Africa. Below is a brief description of the farms included in the study.

1. Commercial layer farms: We included three different layer farms (A, B, C). Two of these are in the south-central part of the provinces of Toledo (39.450527, −3.628713) and Cuenca (39.542977, −1.934466), designated as sites A and B and house 50,000 and 600,000 layers in cages indoors, respectively. The surroundings of these poultry farms primarily consist of fields of non-irrigated crops, including vineyards, barley fields, and almond trees. Additionally, the farms are situated near or include small water sources such as temporary ponds or streams. The third farm designated as site C (39.455741, −2.015767), holds free- range layers and is surrounded by vineyards, barley fields and open pine tree forest. On the premises used by the chickens are almond trees.
2. Red-Legged Partridge Farms: We sampled two different red-legged partridge farms in the north of Ciudad Real (39.232715, −3.602193) and Albacete (38.937303, −2.556022) provinces, designated as sites D and E, respectively. Both farms are situated on the outskirts of a village alongside other agricultural operations. The red-legged partridges raised on these farms are intended for release in hunting estates for recreational hunting and later use in the game meat industry. The entire production cycle, except for the first month of chick rearing, occurs outdoors. This includes housing juvenile partridges in large groups in flight cages and of the breeders in pairs in elevated breeding cages. Like the layer farms, the arable fields surrounding these farms predominantly consist of non-irrigated crops, such as vineyards, cereal plots, almond and olive trees, and open pine forest.

### 2.2 Camera trapping design

We used camera traps Little Acorn CT cameras (Ltl 5310 Series LED IR Invisible) on each of the study farms, to cover at least one of each phenological periods (spring and fall migration, breeding, and wintering) in locations representative of the study farms, particularly in places attractive to birds, such as silos, water points, temporary ponds, as well as at the entrances of poultry houses/poultry enclosures and feeding and watering areas. The number of cameras employed on each farm varied with the size of the farm and camera availability between 5 and 10 cameras. Cameras were deployed to obtain a similar number of days (*n* = 7) of camera trapping on each farm for each phenological period. However due to logistical reasons (distance of farms, camera failures) the number of trapping events varied between farms and phenological periods and the data was corrected according to camera trapping effort. Sampling involved the use of 5–10 cameras that remained active for an average of 18 days on commercial layer farms (Farm A; camera activity range = 3–69 days, total sampling effort = 349 camera-days. Farm B; camera activity range = 6–14 days, total sampling effort = 92 camera-days). The cameras on the pasture-based Farm C were active for an average of 9 days (camera activity range = 6–14 days, total sampling effort = 45 camera-days). On red-legged partridge farms, the cameras on Farm D were active for 6 days (camera activity range = 1–10 days, total sampling effort = 18 camera-days), while on Farm E, the cameras were operational for 9 days (camera activity range = 2–13 days, total sampling effort = 34 camera-days) (Supplementary Table S1).

The camera traps were set up in photo mode with passive motion sensors, capturing three consecutive images every 10 min whenever the motion sensor detected movement within the camera’s field of view. The camera traps were positioned 30–50 cm above ground level with no apparent vegetation obstructions to avoid false detections caused by natural movements such as wind or vegetation. To capture the movement of all birds, regardless of their size, the sensitivity of all cameras was set to high. The cameras operated throughout the day and used infrared flash at night. Each image automatically recorded the date and time. All cameras collected data on SD cards, which were periodically transferred to 4 TB hard drives for storage.

### 2.3 Data management and analysis

All camera trapping (CT) images were examined individually. Only pictures containing birds or other wildlife were included and classified by species. Data extracted from each picture included the following categories: camera location, CET time (day, month, year, hours, minutes), species names, number of visits, taxonomic category order, and migration phenology (spring and fall migration, breeding, and wintering).

We estimated the species richness of wild birds in each study farm using four non-parametric estimators (Abundance-based coverage estimator ACE, incidence based coverage estimator ICE, Chao2, and Bootstrap) with EstimateS v.9.1.0 (28) to assess the species visiting the farms. Two estimators have been used that rely on abundance data and are based on the statistical concept of sampling coverage (ACE and ICE). It refers to the sum of the probabilities of finding observed species within the total of present but unobserved species (29). The ACE estimator makes its estimations considering 10 or fewer individuals per sample, while the ICE utilizes species found in 10 or fewer samples (30). The Chao2 richness estimator combines presence/absence data for a species in a given sample, such as those obtained with camera traps, to estimate whether the species is present and how many times that species is present in the sample set. Finally The Bootstrap estimator was used to assess the variability of the sample. This method involves generating new observations by obtaining multiple samples with replacement from the original sample. Its significance lies in its ability to consistently estimate the sampling distribution of a statistic and to accurately estimate its variance (31).

We used the average of these estimators to calculate the proportion of species documented on the farms, dividing the number of observed species by the mean of the estimators. Additionally, the percentage of registered species is presented as a measure of sampling completeness (%) (Supplementary Table S2). Individual rarefaction curves were calculated using 95% confidence intervals from the estimator (32). To estimate the number of visits by individual wild birds, we classified pictures according to O’Brien et al. (33) into dependent and independent events (Supplementary Figure S2). We designated events as independent when there was a time gap of more than 30 min between two consecutive photos of the same species, or at least two different species were present in the three consecutive images (as illustrated in Supplementary Figure S2A). On the other hand, events were classified as dependent when all three images featured birds of the same species, making it impossible to determine whether the same or a different individual was present in the picture, and when time between two consecutive photos of the same species was less than 30 min (as shown in Supplementary Figure S2B).

To account for the hypothesis of the house sparrow as a bridge species, we investigated the interaction of house sparrows with other species through camera traps. For this we recorded any interaction of the house sparrow, whether direct or indirect, with any other wild bird species. We defined a direct contact as the presence of one or more house sparrow and any other bird species together in the same picture (see Supplementary Figure S2C). Additionally, we considered any image that showed a bird species different from the house sparrow within a period of less than 24 h before capturing an image with house sparrows in the same location as an “indirect contact” (see Supplementary Figure S2D).

Using the data obtained from the camera traps we analyzed factors that modulate bird communities and wild bird visits to poultry farms, as well as direct and indirect contacts between house sparrows and other wild bird species. Specifically, we included explanatory variables such as the type of poultry farm, bird order, phenology, and migratory behavior (Table 1).

**Table 1:** Predictor categories defined for the models used.

Predictor	Description
Poultry farms	Caged layer farms
	Free- range layer farms
	Red legged partridge farms
Bird order	Anseriformes
	Bucerotiformes
	Charadriiformes
	Columbiformes
	Passeriformes
	Pelecaniformes
Migration	Spring migration (February–April)
	Breeding (May–July)
	Fall migration (August–October)
	Wintering (November–January)
Behavior	Resident wild birds
	Migratory wild birds
	Partial migrants

For this analysis, we constructed three generalized linear models (GLM) with a binomial distribution and a logit link function. The first model was used to explore the effect of explanatory variables on wild bird visits, while the second and third models examined their effect on the observation of direct and indirect contacts of other wild bird species with house sparrows. The dependent variable was defined as the count of independent events involving wild birds in the images, encompassing both direct and indirect contacts of wild birds with house sparrows. Model construction followed a stepwise forward Akaike selection (34). Statistical analyses were carried out using SPSS 28.0 (Statistical Package for Social Sciences Inc.), with statistical significance set at *p* < 0.05.

## 3 Results

A total of 139,246 images were captured with the camera traps in the years 2018–2019. Among these, 78,779 images were taken on commercial layer farms, 30,187 on free-range layer farms, and 29,280 on red-legged partridge farms. A total of 31,816 birds belonging to 33 species, 21 families, and seven different orders were observed on the five farms. Out of these, 18 were resident bird species (55%), 11 were partially migratory birds (33%), and three were migratory bird species (12%). Most resident species belonged to the order Passeriformes (72%), such as the house sparrow, tree sparrow, and spotless starling (*Sturnus unicolor*) (see Supplementary Table S3).

The non-parametric estimators calculated 96.4% ± 3.6 and 95.2% ± 4.8% of the total observed species richness on Farms D and E, respectively. However, the non-parametric estimators suggest that species richness is higher on the remaining farms: Farm A (83.8% ± 16.2%), Farm B (69.2% ± 30.8%), and Farm C (55.4% ± 44.6%) (see Supplementary Figure S1, Supplementary Table S2).

**Figure 2:**
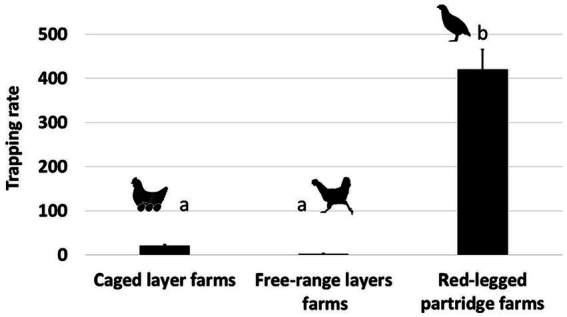
Wild bird trapping rate in each poultry farm (Caged layer farms, free-range layers farms, red-legged partridge farms).

We detected a significantly higher frequency of visits by wild birds on red-legged partridge farms as compared to caged layer farms and free-range layer farms (see Table 2, Figure 2). Visits detected on caged layer farms were less numerous, but from a much larger variety of species (Supplementary Table S3, Supplementary Figure S3). Species in the Columbiformes and Passeriformes order were significantly more likely to be detected (Table 2). Also, the number of bird visits detected was significantly higher during the breeding season (see Table 2, Supplementary Figure S3).

**Table 2:** Results of the GLM used to evaluate the number of visits of wild birds to type of poultry farms, bird order, migration phenology and migratory behavior.

Predictor		*B*	S. Error	*p*-value
Intercept		1.713	0.3285	**<0.001**
Poultry farms	Caged layer farms	–	–	–
	Free-range layer farms	−1.393	0.2061	**0.001**
	Red legged partridge farms	0.902	0.1234	**<0.001**
Bird order	Anseriformes	−0.777	0.5699	0.151
	Bucerotiformes	−2.833	1.1298	0.012
	Charadriiformes	0.822	0.4808	0.087
	Columbiformes	1.586	0.3673	**<0.001**
	Passeriformes	1.539	0.3450	**<0.001**
	Pelecaniformes	–	–	–
Migration	Spring migration	–	–	–
	Breeding	0.733	0.1383	**<0.001**
	Fall migration	−1.505	0.1479	**0.000**
Migratory behavior	Wintering	−1.419	0.1357	**0.000**
	Migratory	−3.836	0.2881	**0.000**
	Partially migratory	−1.769	0.1893	**<0.001**
	Resident	–	–	–

Statistically significant results are highlighted in bold.

Resident bird species visited farms significantly more than partially migratory or migratory species (Table 2). Additionally, resident species had significantly more direct and indirect contacts with house sparrows as compared to migratory species (Table 3).

**Table 3:** Results of the GLM used to evaluate the number of the direct contacts of wild birds with house sparrows according to type of poultry farm, bird order, migration phenology and migratory behavior.

Predictor		*B*	S.Error	*p*-value
Intercept		−0.63	0.6410	0.325
Poultry farms	Caged layer farms	–	–	–
	Free-range layers farms	−29.639	399222.1547	1.000
	Red legged partridge farms	−0.188	0.1762	0.286
Bird order	Anseriformes	−1.235	0.9792	0.207
	Bucerotiformes	–	–	**–**
	Charadriiformes	−0.270	0.7456	0.717
	Columbiformes	0.869	0.6521	0.183
	Passeriformes	0.759	0.6239	0.224
	Pelecaniformes	–	–	–
Migration	Spring migration	–	–	–
	Breeding	0.498	0.1796	**< 0.05**
	Fall migration	−1.683	0.2794	**< 0.01**
Migratory behavior	Wintering	−1.441	0.2605	**< 0.01**
	Migratory	−2.546	0.6183	**<0.001**
	Partially migratory	−0.315	0.2881	0.274
	Resident	–	–	–

Statistically significant results are highlighted in bold.

The house sparrow was the most frequently captured species in photographs, on all five farms and during all phenological periods. Spotless starlings were observed on four farms, being more abundant in the red-legged partridge farms, but not on the free-range layer farm (Supplementary Figure S4). In three of the farms, the camera traps recorded species such as the white wagtail (*Motacilla alba*), crested lark (*Galerida cristata*), and rock pigeon (*Columba livia*) (Supplementary Table S3). On the free-range layer farm, the house sparrow was less common (11%), and the Eurasian magpie (*Pica pica*) was the most frequently observed species (53%) (Supplementary Figure S4). Notably although anecdotical, waterbirds (mallard *Anas platyrhinchos*, black winged stilt *Himantopus himantopus*, ring-necked plover *Charadrius hiatus*) were detected on at least three of the farms (Supplementary Table S3).

Direct contacts between house sparrows and other species were significantly more likely during the breeding season and with resident bird species (see Table 3, Figure 2). The need to seek food and water, especially in juvenile birds, increases interactions with other species, particularly with the house sparrow. Indirect contacts with house sparrows (use of the same location by house sparrows within a time span of 24 h and thus potential of exposure to fecal contamination) was significantly more likely during the breeding season and least likely between house sparrows and Anseriformes and on the free-ranger layer farm (Table 4). Species that interacted more frequently with the house sparrow belonged to the order Passeriformes, being almost twice as common as the second most common type, the Columbiformes. Birds in the Charadriiformes order had fewer contacts with the house sparrow (see Supplementary Figure S4).

**Table 4:** Results of the GLM used to evaluate the number of the indirect contact of wild birds with house sparrows in relation to type of poultry farm, bird order, migration phenology and migratory behavior.

Predictor		*B*	S.Error	*p*-value
Intercept		−0.459	0.5376	0.393
Poultry farms	Caged layer farms	–	–	**–**
	Free-range layers farms	−2.392	0.4451	**<0.001**
	Red legged partridge farms	−0.611	0.1786	<0.001
Bird order	Anseriformes	−2.528	1.1509	**0.022**
	Bucerotiformes	–	–	**–**
	Charadriiformes	−1.042	0.6605	0.115
	Columbiformes	0.638	0.5506	0.246
	Passeriformes	0.471	0.5181	0.363
	Pelecaniformes	–	–	–
Migration	Spring migration	–	–	–
	Breeding	0.176	0.1791	0.326
	Fall migration	−1.363	0.2325	**<0.001**
Migratory behavior	Wintering	−1.700	0.2540	**<0.001**
	Migratory	−1.884	0.4288	**<0.001**
	Partially migratory	−0.035	0.2487	0.889
	Resident	–	–	**–**

Statistically significant results are highlighted in bold.

## 4 Discussion

Our study applies camera trapping technology to the poultry farm environment to comparatively describe the composition and seasonal changes of farm bird communities on different types of layer/gamebird farms. This is to the best of the authors knowledge the first time such a study is carried out in Spain. Previous work and data collected in the present study provide evidence of the house sparrow as key species that enters layer buildings and flight cages of partridges and other bird species which has led to its designation, together with several other synanthropic bird species as potential bridge hosts (6, 9, 35) (Supplementary Figure S5). However, although bidirectional exchange of pathogens at the interface has been demonstrated and the potential of bridge hosts is generally accepted (36) little information exists yet on the frequency of contact and potential of contamination of resident farm birds by visiting migratory birds. For this reason we used the pictures obtained to also study the contact of wild birds that visit farm premises, but are unlikely to enter the buildings and enclosures, with the house sparrow (4, 5, 21).

The farms studied here are not directly connected to any wetland, which makes them theoretically unattractive to wild waterfowl (13), however satellite telemetry data has recently shown that waterfowl occasionally does make incursions onto poultry farm premises (23) and in fact our data shows, that even the studied farm premises are occasionally visited by waterbirds, either at open water tanks or temporary pools after heavy rains (Supplementary Figure S5).

The camera traps used for this study covered locations at the external fencing of the farms, aggregation hotspots such as water and food sources and possible entrances to the farm buildings. Actual farm bird diversity is certainly greater than that observed in this study. Camera traps do not always capture the total number of bird species visiting the farm, as they are positioned and focused on specific points, making it challenging to obtain a complete picture of the bird population. Additionally, even with increased sampling effort, as observed in farms A and B (*n* = 349 days; *n* = 92 days), we notice that the observed richness does not align with the studied estimators, indicating the need to extend our sampling period (Supplementary Table S2, Supplementary Figure S1). Logistical issues such as farm size, the number of cameras used, or limitations in data storage due to an abundance of individuals in a single location hinder obtaining the actual number of species. This is due to the size of the poultry farms, surrounding vegetation and habitats (various crops, buildings) (24) and changes in these (crop harvest, sowing etc.). However as the camera trap distribution design was similar for all studied farms and data was corrected for trapping effort, we can at least to some extend compare the collected information (21). Our results show that red-legged partridge farms attracted the highest number of visits, followed by caged and free-range layer farms. Most of the observed species were passerines. Possible reasons why wild birds were most attracted to partridge farms are the relatively easy access to food and water as both breeders and juvenile partridges are raised outdoors in batteries of breeding cages or large flight cages, respectively. Previous studies have shown that wild birds are not particularly attracted to free-range chicken farms, potentially as the grazing areas are rapidly degraded by the chickens while the large numbers of chickens also appear to intimidate most wild birds (24).

Among the species identified, the house sparrow is the most frequently observed and interacts directly and indirectly with a large variety of other species and a considerable number of individuals from which they could acquire pathogens including AIV as sparrows have been shown to be susceptible to AIV infection (18, 37, 38). The adaptation of the sparrow to human modified habitats, gregarious behavior and obligate commensalism drives their potential as bridge host (39). Other frequently detected species included the spotless starling and the rock dove. Both species are known to be frequently exposed to and carriers of pathogens such as Salmonella spp. and antibiotic resistance mechanism carrying *Escherichia coli* among others (10). Also, European starlings, a species closely related to the spotless starling have been experimentally shown to be able to transmit avian influenza virus to poultry (40). Species detected in free-range layer farms such as the white wagtail are consistent with the species detected in a study of free-living birds in enclosures of duck farms in France (5), while other species observed on the duck farms, such as cattle egrets, were observed less frequently.

**Figure 3:**
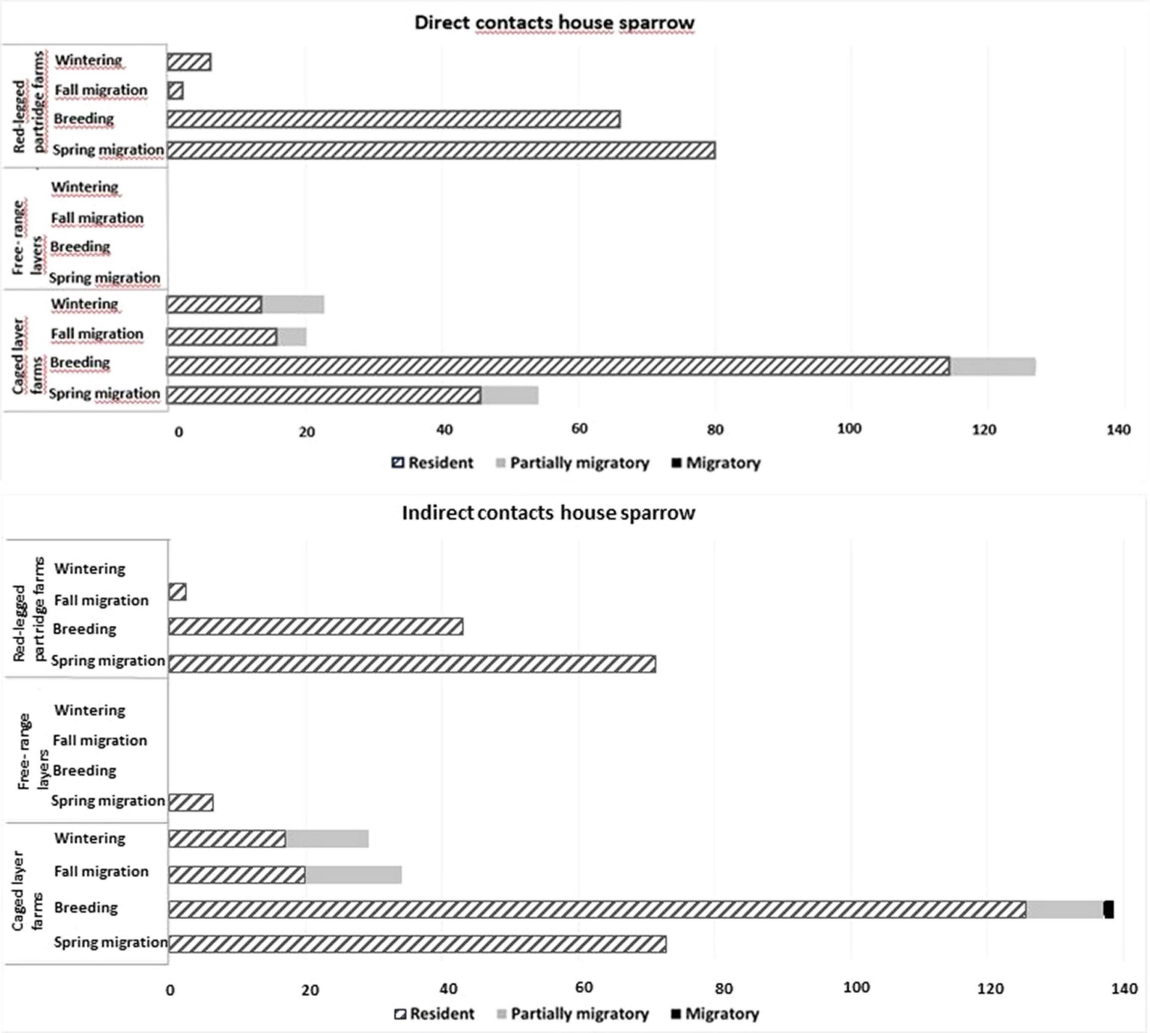
Total number of direct (in black) and indirect (in gray) contacts of visiting wild birds with house sparrows according to their migratory behavior (resident, partially migratory, migratory).

Direct but also indirect contacts are often the main risk factor in pathogen transmission between wild and domestic birds (41). Contacts of other birds with house sparrows were observed generally in association to food and/or water and less frequently roosting space. Our results show that direct and indirect contacts of sparrows with other bird species on farms occur significantly more frequently with resident species than with partially or fully migratory species (Figure 3). If, in addition to direct contacts, the possibility of indirect contamination through secreta and feces is considered, with a maximum residence time of approximately 24 h, the potential for contamination of a sparrow by AIV or other pathogens doubles (Figure 3).

The highest number of direct and indirect contacts were recorded during the breeding season, followed by the spring and fall migration periods. For the wintering season, hardly any direct and indirect contacts were recorded even though the highest number of visits occurred at this time of year. As our farms are situated in a Mediterranean continental climate where food and water in natural habitats are more restricted in summer than in winter, likely during this period the availability of food and water was less important than other functions of the farm environment. More frequent farm visits during the breeding season, may be linked to the high number of juvenile individuals that rely on easily accessible resources. The high number of juvenile individuals increases the number of contacts with likely a higher number of naïve, more susceptible individuals thus increasing the probability of pathogen transmission (42). Also, during the breeding and post-breeding periods, the food requirements of breeding adult sparrows are at the highest, while it is under the Mediterranean continental climate the period with less food and water resources increasing the attraction to farm premises considerably. The lower number of direct and indirect contacts during fall migration is probably due to the abundance of food (cereal and fruits) in the season.

Both migratory and resident birds can be carriers of pathogens either directly or by exposure in contaminated environments and by Borie et al. (43) by contaminating resources, such as water or feed, with their droppings. Here we have detected few migratory or partially migratory birds on farm visits which in turn underlines the potential bridge host role of sparrows (18). While contact restriction measures are generally focused on the protection of poultry they also need to take into account the risk of environmental transmission from poultry to wildlife, by sewage, feathers, dust and aerosols from that can represent a major source of contamination for synanthropic wild birds such as the house sparrow that could than contaminate non-resident visiting birds during direct or indirect contacts (44).

## 5 Conclusion

Camera trap-based characterization allowed to describe composition and fluctuation of wild bird communities in different farm type environments across seasons and to identify species that could represent bridge hosts. Identifying the house sparrow as key species with resident populations on farms we evidence its connection to other bird species that visit the farm environment. Our results indicate that general biosecurity measures such as restriction o access to food and water are highly effective as the number of bird visits on red-legged partridge farms were significantly more frequent, than on other farms.

## Data availability statement

The original contributions presented in the study are included in the article/Supplementary material, further inquiries can be directed to the corresponding author.

## Author contributions

AS-C: Formal analysis, Methodology, Visualization, Writing – original draft, Writing – review & editing. M-CC: Methodology, Resources, Writing – review & editing. YR: Methodology, Resources, Writing – review & editing. TC: Methodology, Resources, Writing – review & editing. UH: Conceptualization, Methodology, Resources, Writing – original draft, Writing – review & editing.
