# Supplementary material for: Seasonal changes in bird communities on poultry farms and house sparrow—wild bird contacts revealed by camera trapping
Source: Front Vet Sci. 2024 Feb 20;11:1369779. doi: 10.3389/fvets.2024.1369779 (PMC10912304; doi:10.3389/fvets.2024.1369779)
Supplement: Supplementary file 1 [file Data_Sheet_1.docx]

***Supplementary Material***

**Seasonal changes in bird communities on poultry farms and house sparrow - wild bird contacts revealed by camera trapping.**

Alberto Sánchez-Cano^*^, María Cruz Camacho Sánchez-Camacho, Yolanda Ramiro, Teresa Cardona Cabrera, Ursula Höfle

***Correspondence:** Alberto Sánchez-Cano: albertosanchezcano91@gmail.com

**Supplementary information**

**Table S1**. Summary of camera trap sampling results. The letters A, B, C correspond to commercial layer farms, and E, D to red-legged partridge farms. The total number of camera traps used for each farm is detailes, along with the sampling effort and camera activity range.

| **Layer/Poultry farms** | **ID Farm** | **Total cameras** | **Sampling effort in days** | **Camera activity range** |
| --- | --- | --- | --- | --- |
| Caged layers farms | A | 10 | 349 | 3- 69 days |
|  | B | 10 | 92 | 6-14 days |
| Free-range layers | C | 5 | 45 | 6-14 days |
| Red-legged partridge farms | D | 5 | 18 | 1-10 days |
|  | E | 5 | 34 | 2-13 days |

**Table S2.** Observed and expected richness of wild bird species in each study farm based sampling records. The letters A, B, C correspond to commercial layer farms, and E, D to red-legged partridge farms.

| Farm | Observed richness | Nonparametric richness estimator ^a^ | | | |  |  |  |
| --- | --- | --- | --- | --- | --- | --- | --- | --- |
|  |  | ACE | ICE | Chao2 | Bootstrap | Mean | SD | % |
| A | 25 | 29.36 | 34.39 | 31.6 | 28.74 | 29.8 | 2.8 | 83.8 |
| B | 16 | 17.74 | 29.97 | 32.5 | 19.34 | 23.1 | 7.0 | 69.2 |
| C | 9 | 10.3 | 31.68 | 18.63 | 11.61 | 16.2 | 10.2 | 55.4 |
| D | 5 | 5 | 5.51 | 5 | 5.43 | 5.2 | 0.3 | 96.4 |
| E | 4 | 4 | 4.58 | 4 | 4.42 | 4.2 | 0.3 | 95.2 |

**^a^** The four non-parametric estimators are based on species occurrence (presence/absence): average-based species estimator (ACE), incidence-based species estimator (ICE), Chao2 and Bootstrap. The mean (±SD) of the four estimators and the percentage of species recorded (species observed/mean of the estimator x 100) are also shown as a measure of sampling completeness (%)

**Table S3.** Results of camera trapping study on farms. Species grouping and intensity of the capture effort until the initial sighting of the species (trapping rates): Visits of wild birds on the five farms detected by camera traps after correction for trapping effort, categorized by members of the orders (Passeriformes, Anseriformes, Bucerotiformes, Charadriiformes, Columbiformes, Pelecaniformes, Apodiformes), families of wild birds, and migratory behavior (migratory, partial migratory and resident). Farm A, B correspond to caged layers farms, C correspond to free-range layers, and farm E, D correspond to red-legged partridge farms. En each of the farms, the total richness of observed species is noted.

| **Order** | **Families** | **Species (Scientific Name)** | **Migratory behavior** | **Poultry A** | **Poultry B** | **Poultry C** | **Poultry D** | **Poultry E** | **Nº poultry** |
| --- | --- | --- | --- | --- | --- | --- | --- | --- | --- |
| Anseriformes | Anatidae | *Anas platyrhynchos* | Partial migrant | 0.006 |  | 0.267 |  |  | 2 |
| Apodiformes | Apodidae | *Apus apus* | Migratory | 0.001 |  |  |  |  | 1 |
| Bucerotiformes | Upupidae | *Upupa epops* | Partial migrant | 0.003 |  |  |  |  | 1 |
| Charadriformes | Scolopacidae | *Actitis hypoleucos* | Partial migrant |  | 0.098 |  |  |  | 1 |
| Charadriformes | Charadriidae | *Charadrius dubius* | Partial migrant |  | 0.100 |  |  |  | 1 |
| Charadriiformes | Burhinidae | *Burhinus oedicnemus* | Resident | 0.003 |  |  |  |  | 1 |
| Charadriiformes | Recurvirostridae | *Himantopus himantopus* | Partial migrant |  | 0.130 |  |  |  | 1 |
| Columbiformes | Columbidae | *Columba livia* | Resident | 0.011 | 0.935 |  | 84.853 |  | 3 |
| Columbiformes | Columbidae | *Streptopelia decaocto* | Resident | 0.178 |  |  |  |  | 1 |
| Columbiformes | Columbidae | *Columba palumbus* | Resident | 0.001 |  |  |  |  | 1 |
| Passeriformes | Hirundinidae | *Delichon urbicum* | Migratory | 0.003 |  |  |  |  | 1 |
| Passeriformes | Hirundinidae | *Hirundo rustica* | Migratory | 0.009 |  |  |  |  | 1 |
| Passeriformes | Motacillidae | *Anthus campestris* | Migratory | 0.006 |  |  |  |  | 1 |
| Passeriformes | Motacillidae | *Motacilla alba* | Partial migrant | 0.140 | 1.565 | 0.044 |  |  | 3 |
| Passeriformes | Aludidae | *Galerida cristata* | Resident | 0.049 | 0.109 |  | 0.824 |  | 3 |
| Passeriformes | Muscicapidae | *Phoenicurus ochruros* | Partial migrant | 0.014 |  |  |  |  | 1 |
| Passeriformes | Corvidae | *Corvus corax* | Resident | 0.003 |  |  |  |  | 1 |
| Passeriformes | Corvidae | *Pica pica* | Resident | 0.198 |  | 1.022 |  |  | 2 |
| Passeriformes | Emberizidae | *Miliaria calandra* | Resident |  |  | 0.022 |  |  | 1 |
| **Order** | **Families** | **Species (Scientific Name)** | **Migratory behavior** | **Poultry A** | **Poultry B** | **Poultry C** | **Poultry D** | **Poultry E** | **Nº poultry** |
| Passeriformes | Sturnidae | *Sturnus unicolor* | Resident | 0.991 | 11.076 |  | 218.559 | 3.667 | 4 |
| Passeriformes | Passeridae | *Passer domesticus* | Resident | 0.100 | 1.739 | 0.489 | 5.118 | 105.000 | 5 |
| Passeriformes | Passeridae | *Passer montanus* | Resident | 3.295 | 0.076 |  |  |  | 2 |
| Passeriformes | Fringillidae | *Carduelis Carduelis* | Resident | 0.003 |  |  | 0.059 |  | 2 |
| Passeriformes | Fringillidae | *Chloris chloris* | Resident | 0.020 |  |  |  |  | 1 |
| Passeriformes | Fringillidae | *Linaria cannabina* | Resident | 0.003 |  |  |  |  | 1 |
| Passeriformes | Fringillidae | *Serinus serinus* | Resident | 0.040 |  |  |  |  | 1 |
| Passeriformes | Aludidae | *Alauda arvensis* | Partial migrant |  |  | 0.244 |  |  | 1 |
| Passeriformes | Motacillidae | *Motacilla cinerea* | Partial migrant |  |  | 0.156 |  |  | 1 |
| Passeriformes | Phylloscopidae | *Phylloscupus collybita* | Partial migrant |  |  | 0.133 |  |  | 1 |
| Passeriformes | Muscicapidae | *Erithacus rubecula* | Partial migrant |  |  | 0.022 |  |  | 1 |
| Passeriformes | Turdidae | *Turdus merula* | Resident |  |  |  |  | 1.667 | 1 |
| Passeriformes | Corvidae | *Cyanopica cyanus* | Resident |  |  |  |  | 0.001 | 1 |
| Pelecaniformes | Ardeidae | *Bubulcus ibis* | Resident | 0.100 |  |  |  |  | 1 |
| Richness | | | | 23 | 9 | 9 | 5 | 3 |  |


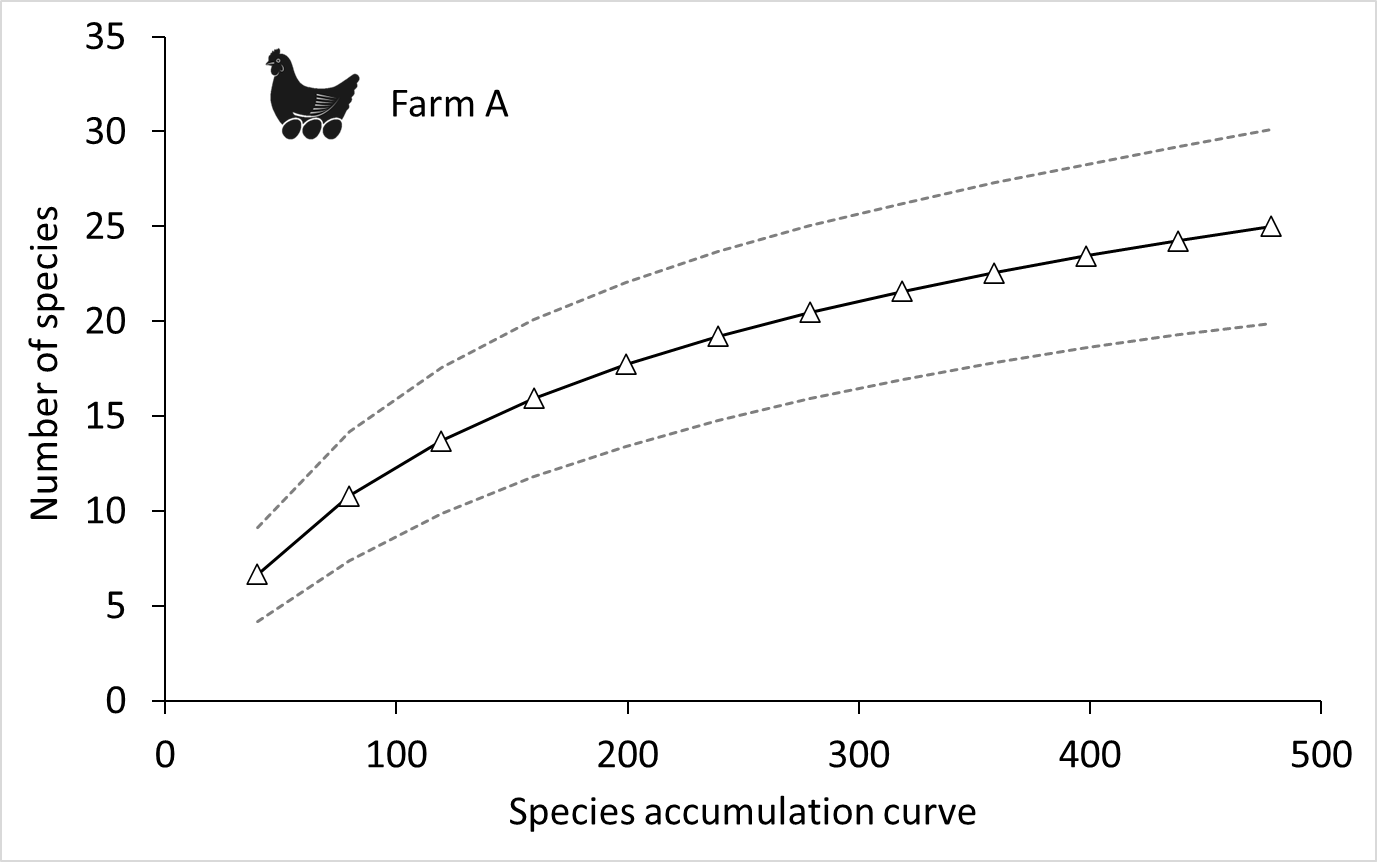


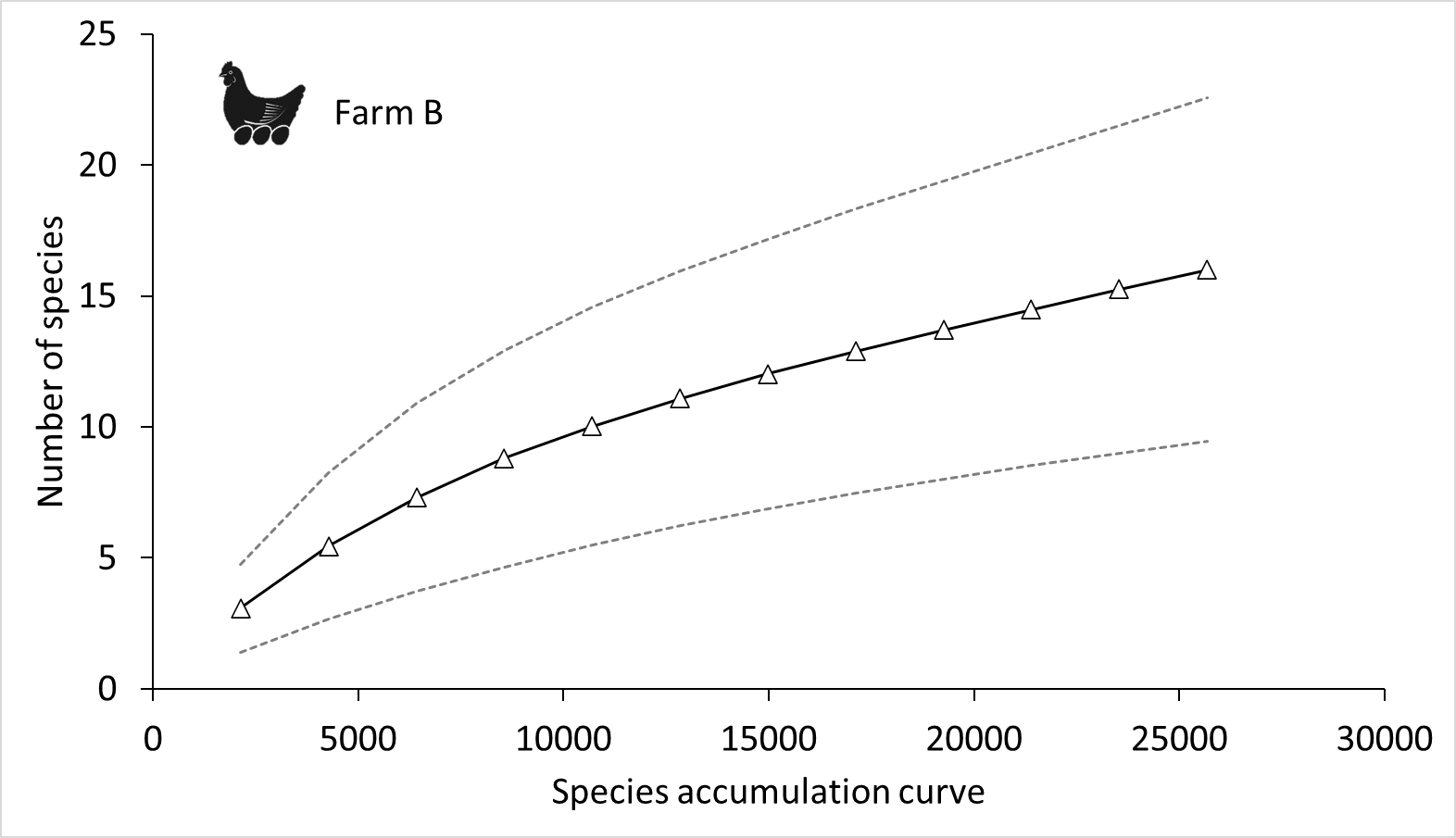


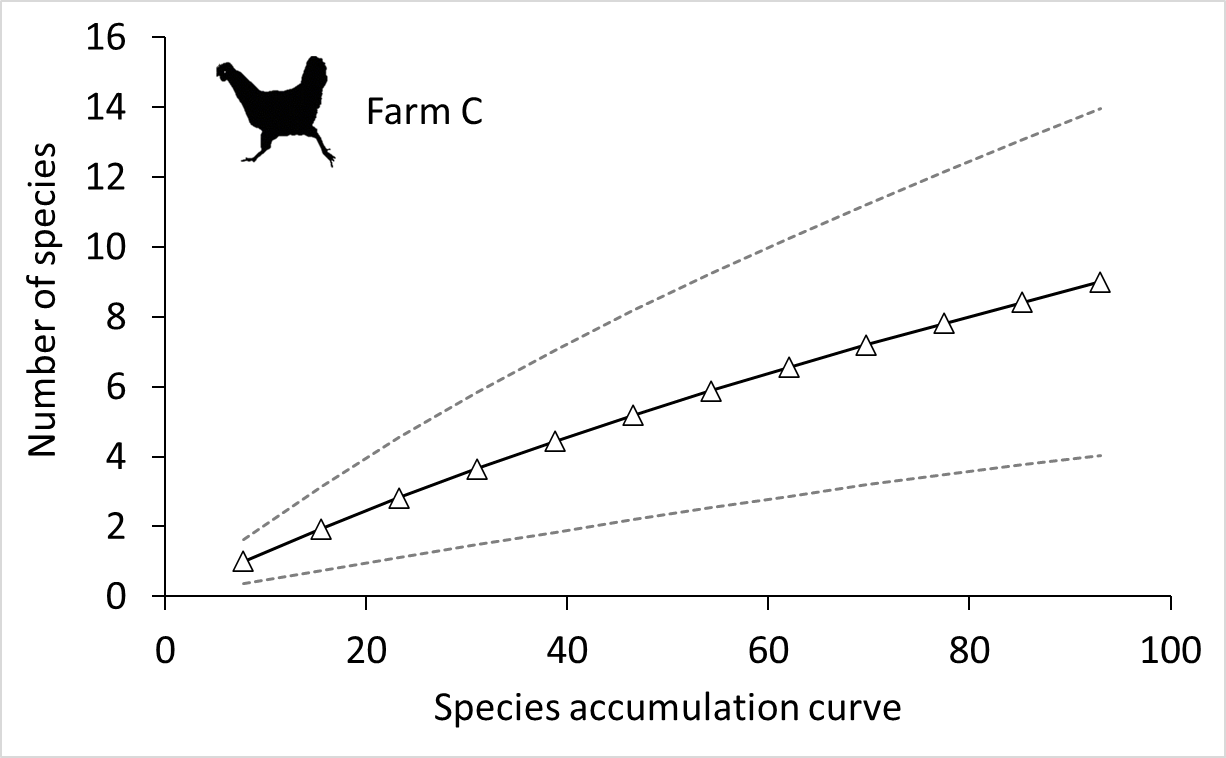


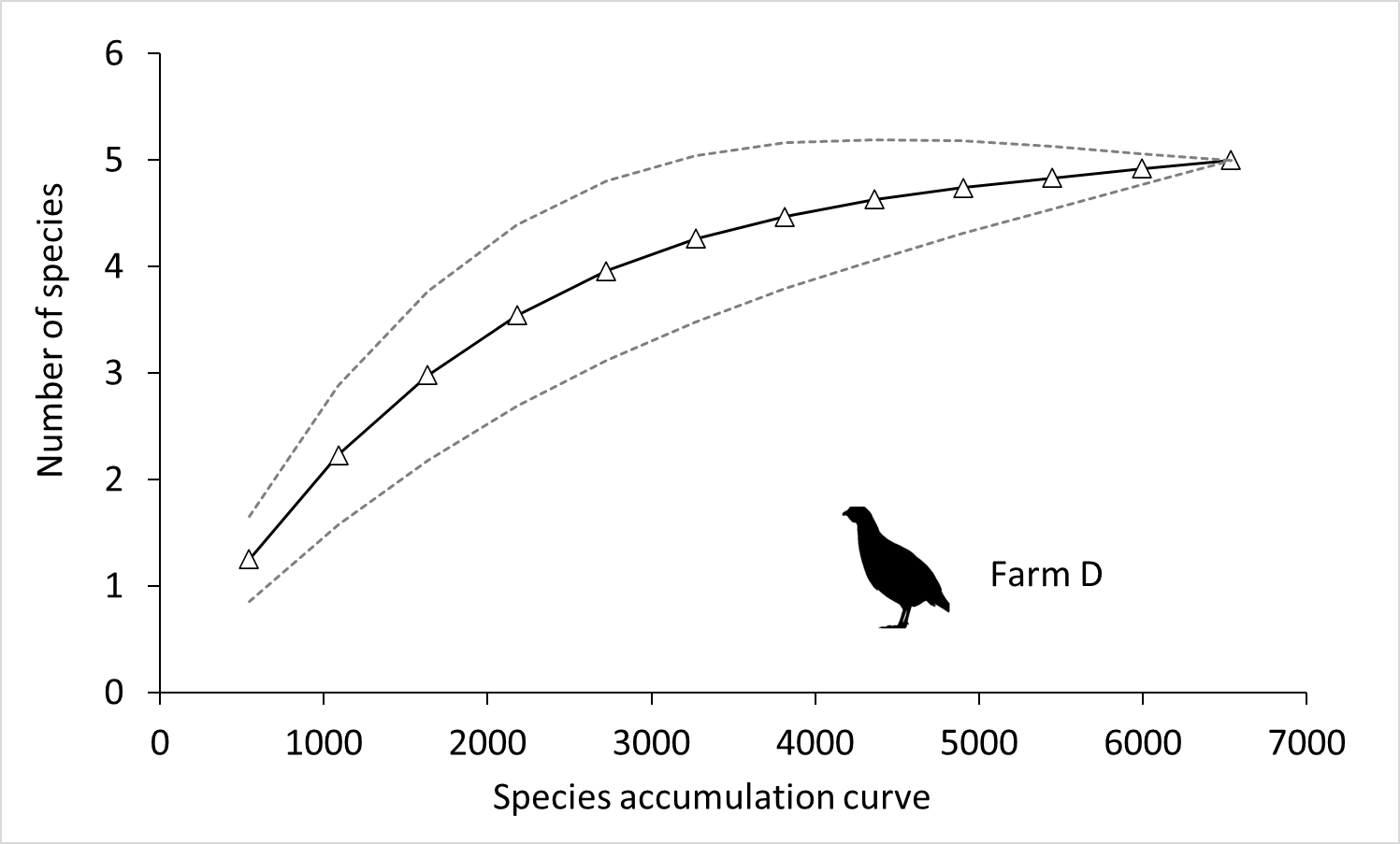


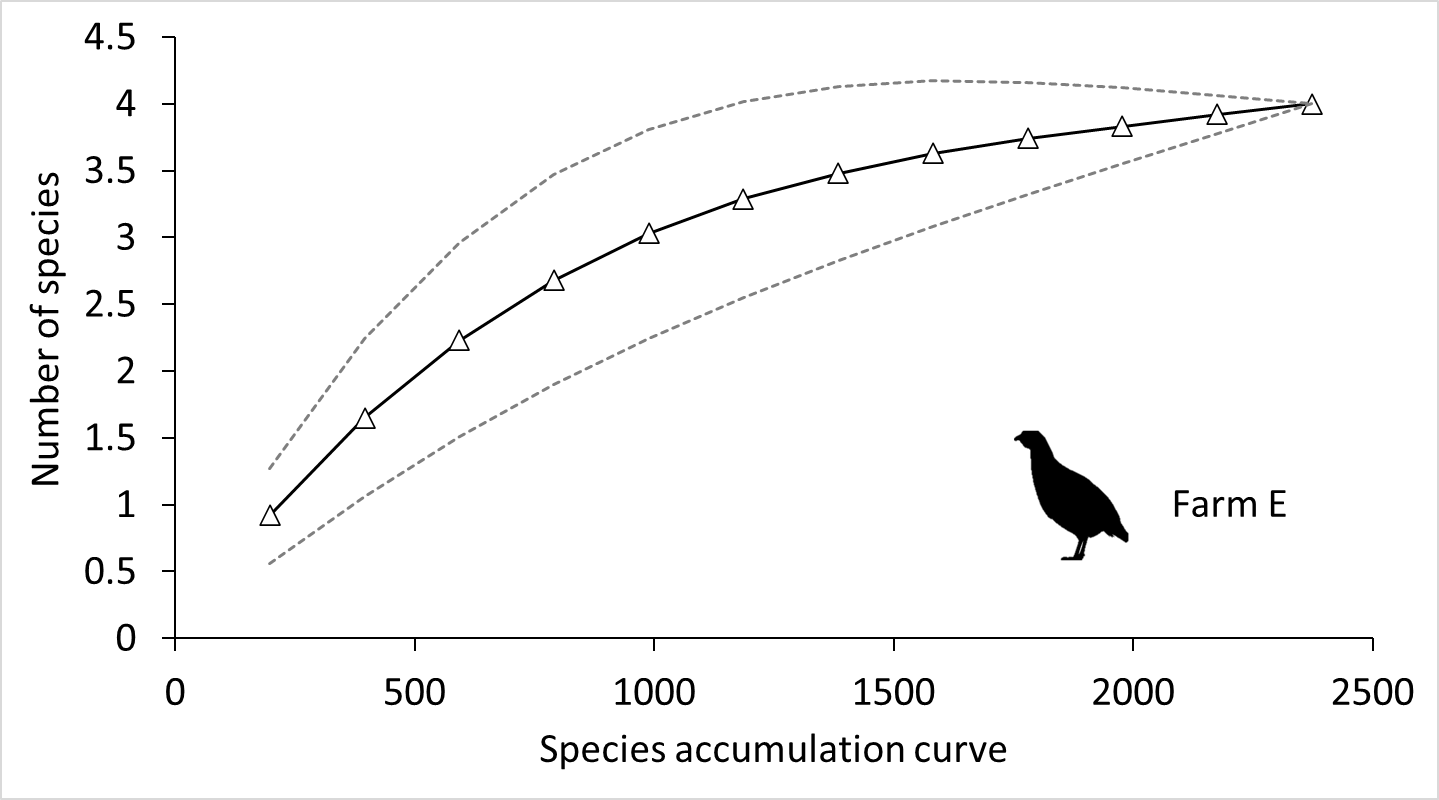


**Figure S1.** Estimated species accumulation curve for each farm under study. The letters (A, B) represent caged layer farms, the letter C denotes the free-range chicken farm, and the letters (E, D) indicate red-legged partridge farms. The central curve is the species accumulation curve. The dashed lines indicate 95% confidence intervals.


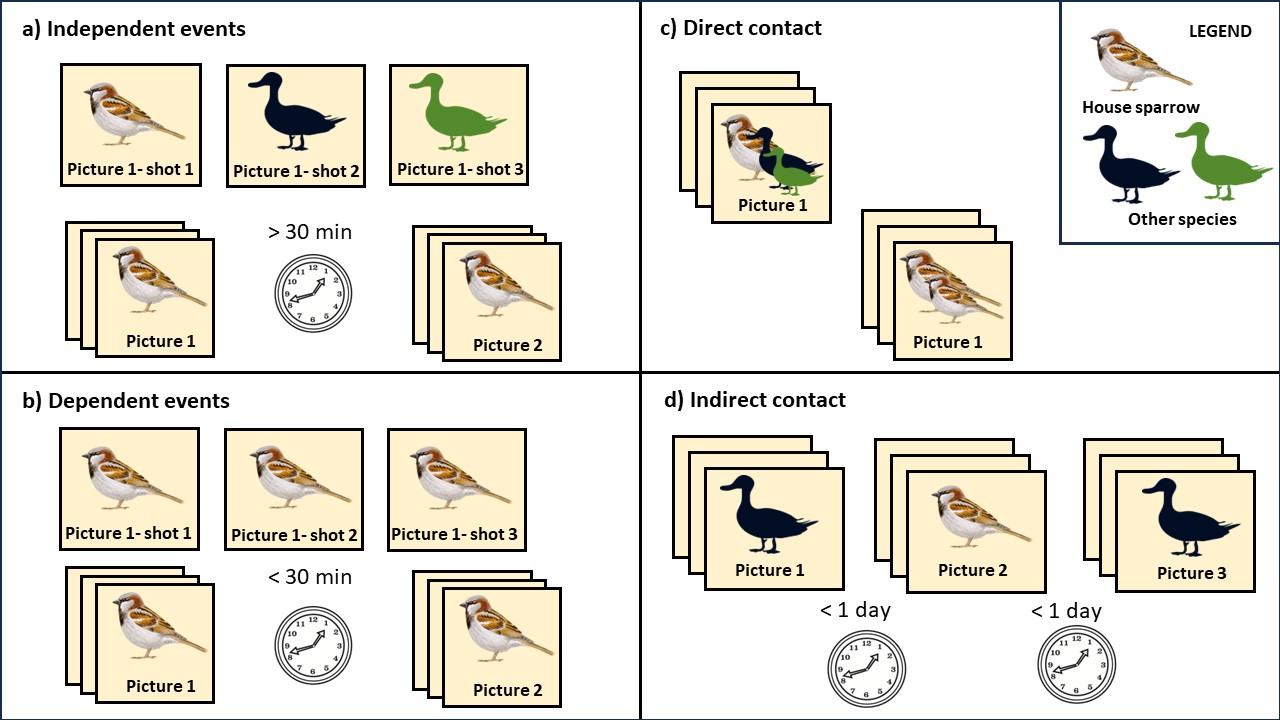


**Figure S2.** Schematic of the methodology of camera trap data analysis: a and b classification of individuals on pictures to ensure individuals on pictures are not counted twice and b and d definition of direct and indirect contact of other bird species with the house sparrow; a) Independent events, b) Dependent events, c) Direct contacts, d) Indirect contacts. For more information, see the main text.

**
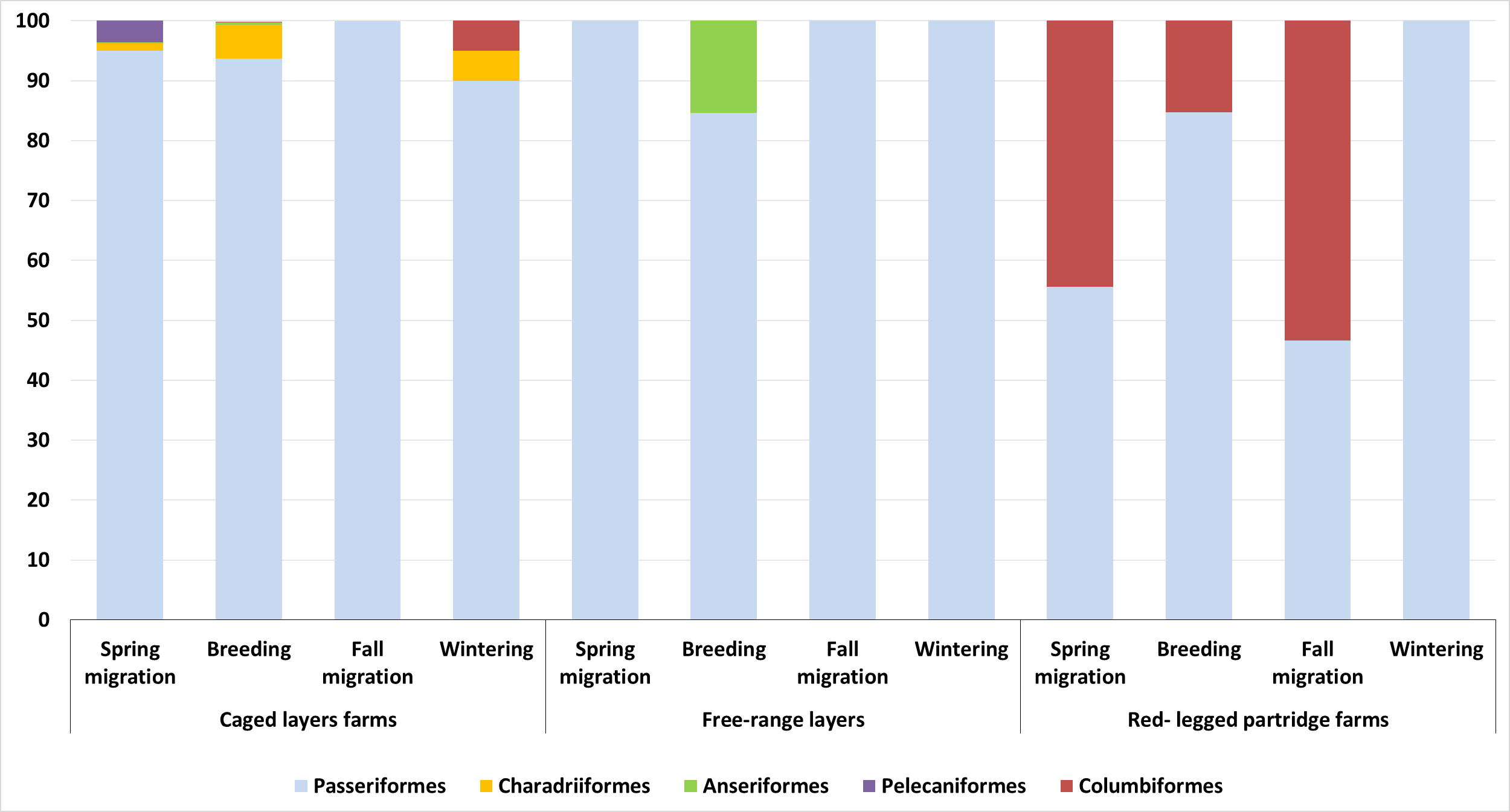
**

**Figure S3.** Proportion of wild bird orders among the total of recorded visits in relation to farm type and phenological season.

**
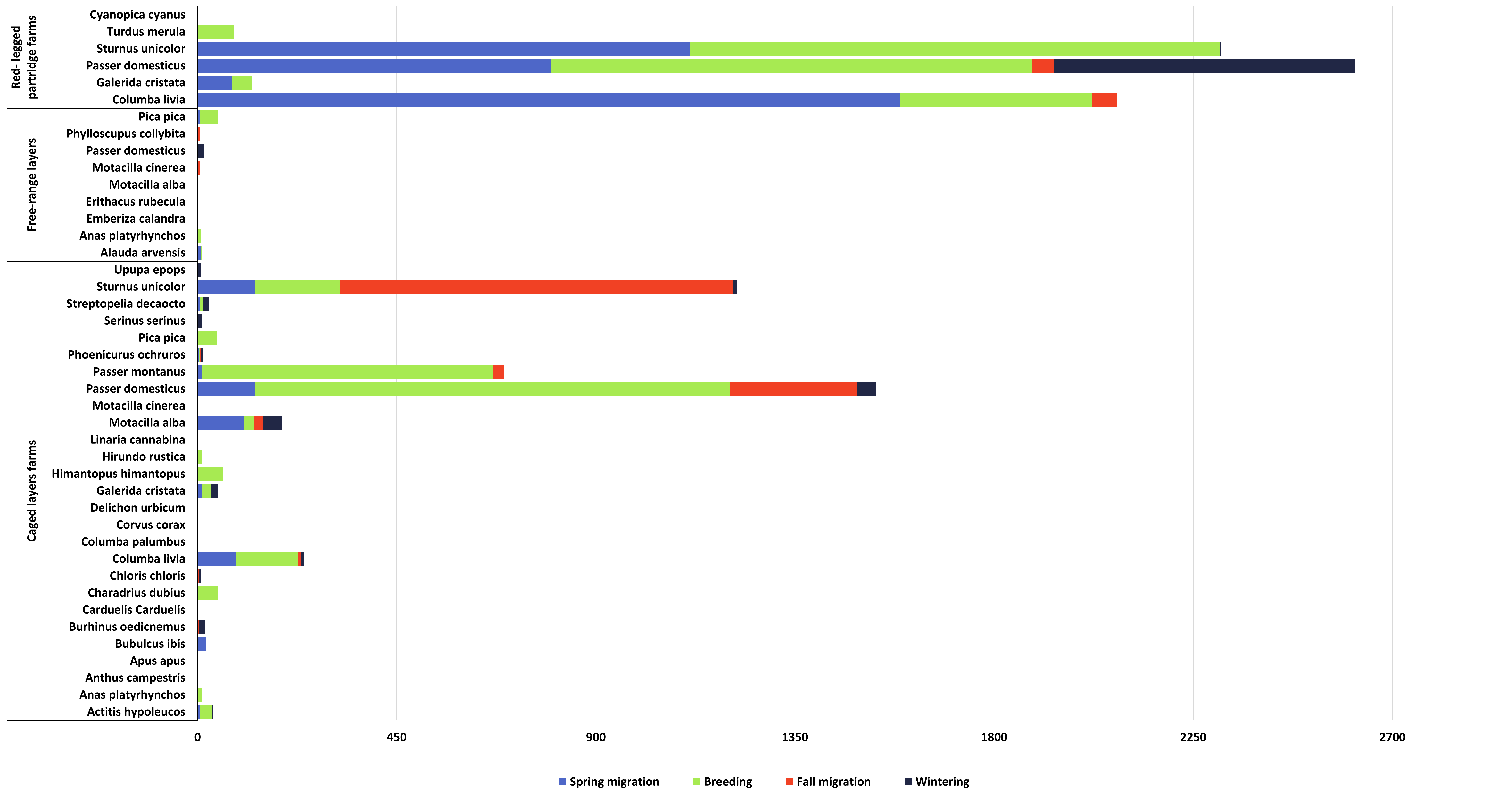
**

**Figure S4.** Wild birds photographed according to phenological period and farm type. Less species but more individuals were detected on red-legged partridge farms.


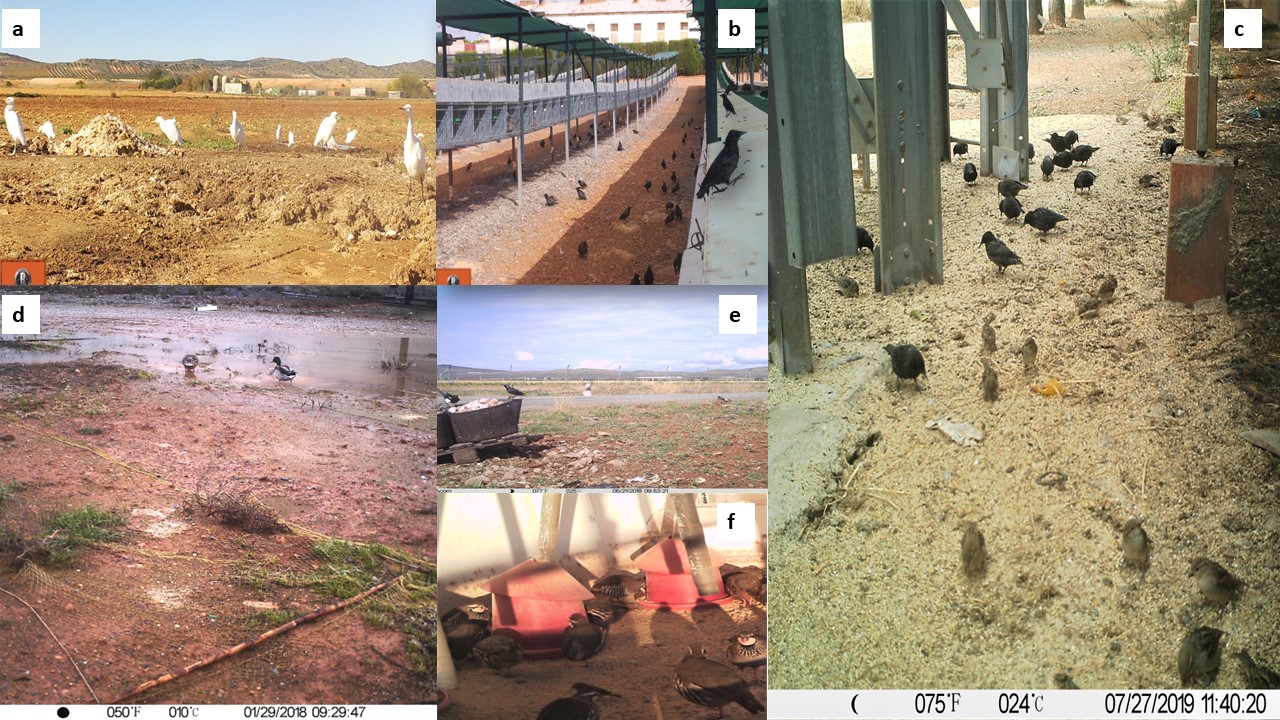


**Figure S5.** Examples of camera trap pictures of some of the species identified at the study sites: a) Cattle egret *(Bubulcus ibis)* b) Spotless starling *(Sturnus unicolor)* c) *Sturnus unicolor* and house sparrow *(Passer domesticus)*, d) Mallard *(Anas platyrhynchos)*, e) *Sturnus unicolor* and magpie *(Pica pica)*, f) Red legged partridge *(Alectoris rufa)* and *Passer domesticus.*


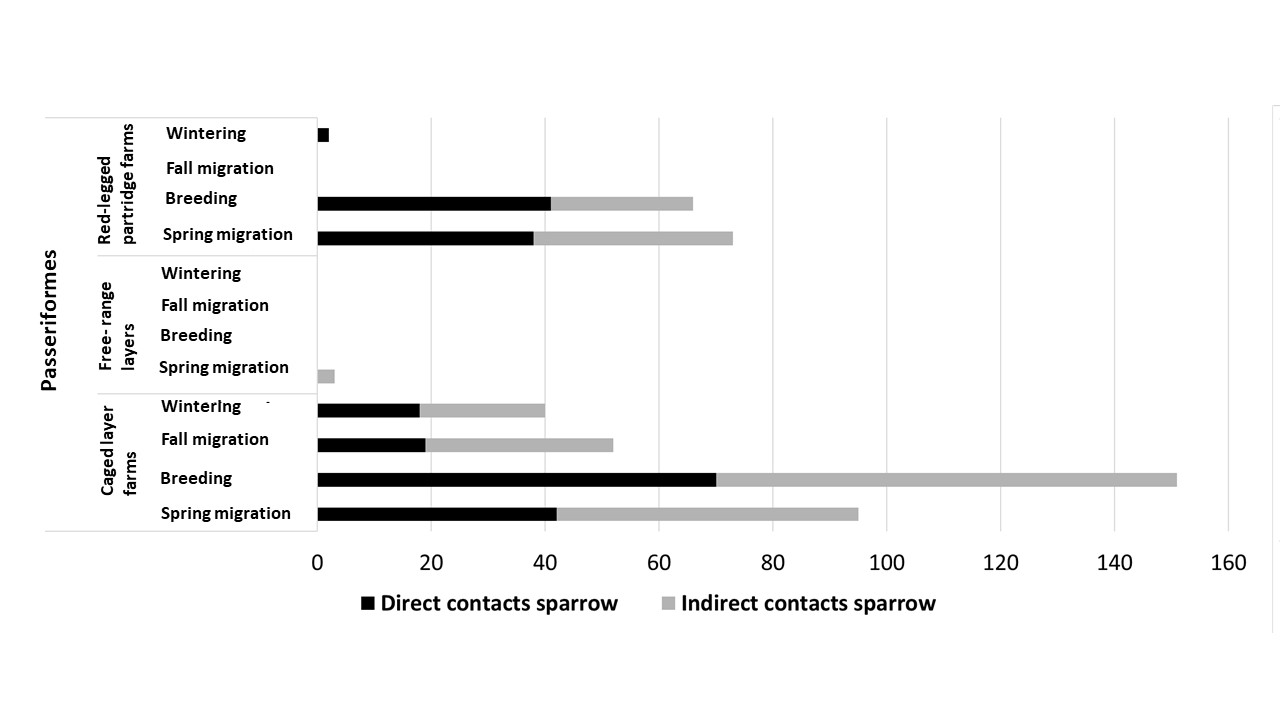


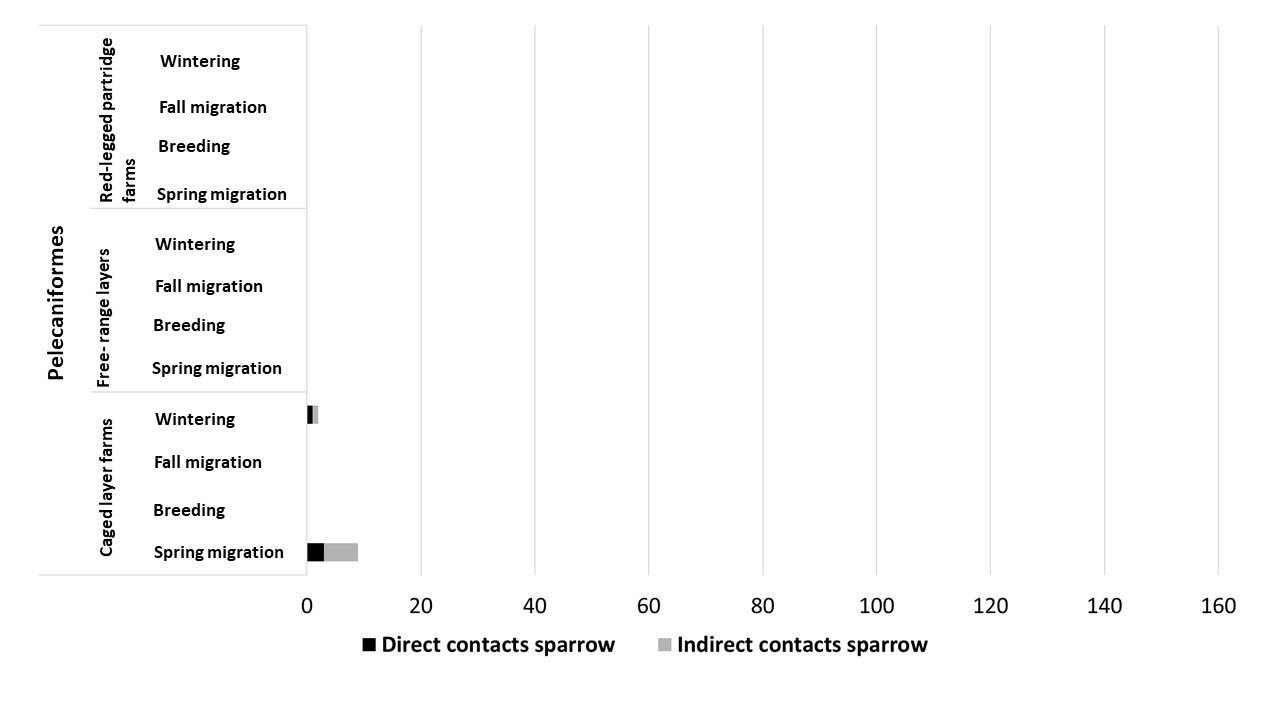


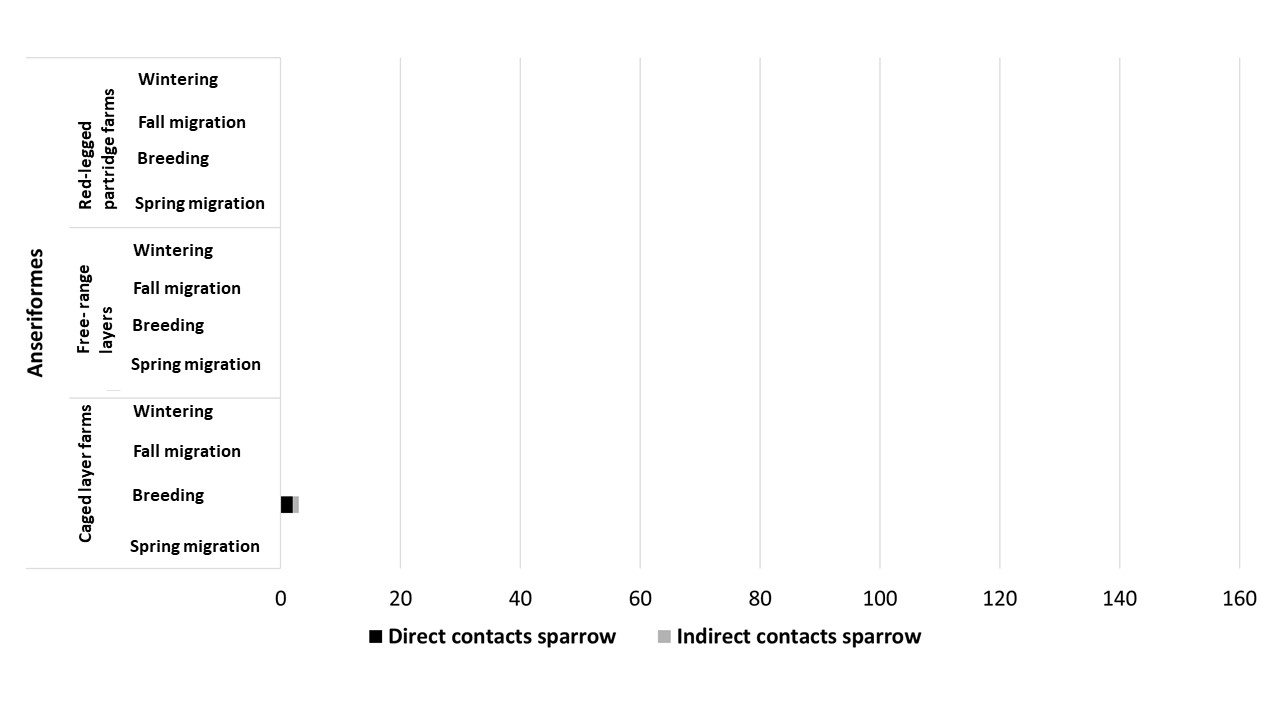


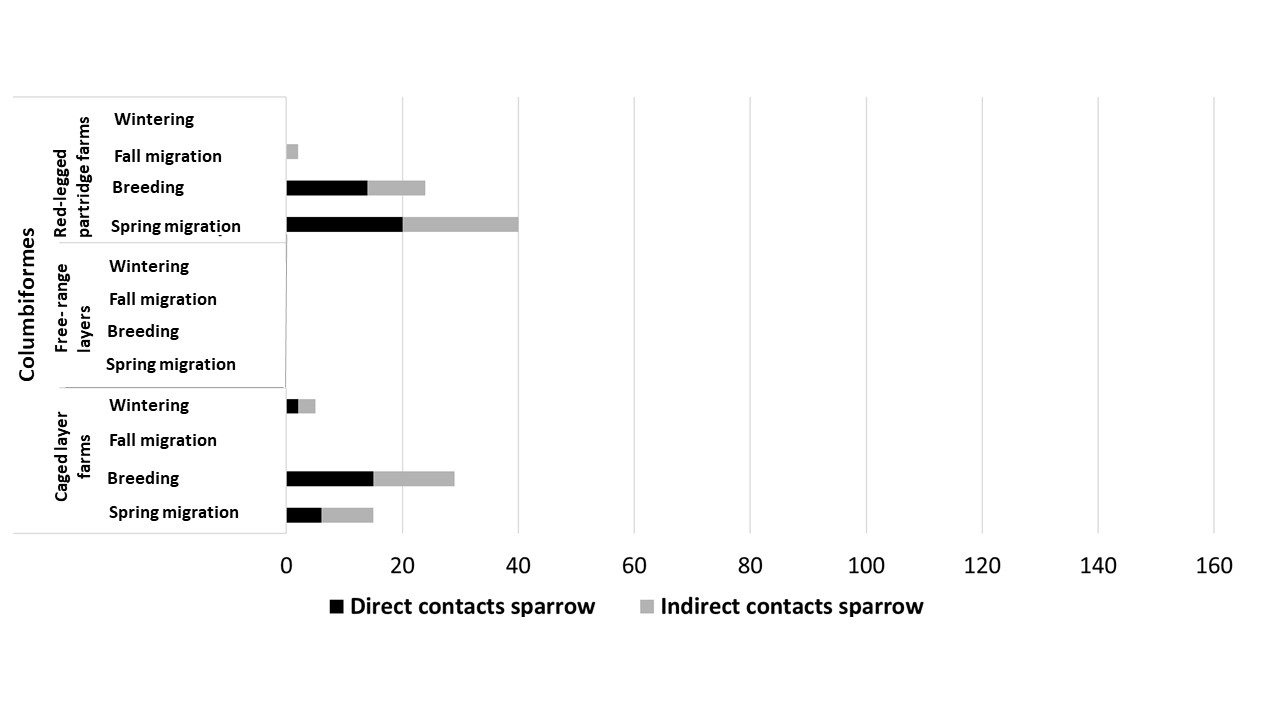


**Figure S6.** Total number of direct (in black) and indirect (in grey) contacts of wild birds of different orders with house sparrows according to farm type and phenological season.
